# Spectinomycin resistance in *Lysobacter enzymogenes* is due to its rRNA target but also relies on cell-wall recycling and purine biosynthesis

**DOI:** 10.3389/fmicb.2022.988110

**Published:** 2022-08-31

**Authors:** Menghao Yu, Youfu Zhao

**Affiliations:** ^1^Department of Crop Sciences, University of Illinois at Urbana-Champaign, Urbana, IL, United States; ^2^Shenzhen Key Laboratory of Synthetic Genomics, Guangdong Provincial Key Laboratory of Synthetic Genomics, Shenzhen Institute of Synthetic Biology, Shenzhen Institute of Advanced Technology, Chinese Academy of Sciences, Shenzhen, China; ^3^Department of Plant Pathology, WSU-IAREC, Prosser, WA, United States

**Keywords:** *Lysobacter*, intrinsic resistance, spectinomycin, rRNA, *mltB*, *purB*

## Abstract

Resistance to spectinomycin emerged after widely used for treatment of gonorrhea. Previous studies revealed that *Lysobacter enzymogenes* strain C3 (LeC3) exhibited elevated level of intrinsic resistance to spectinomycin. In this study, we screened a Tn5 transposon mutant library of LeC3 to elucidate the underlying molecular mechanisms of spectinomycin resistance. Insertion sites in 15 out of 19 mutants recovered with decreased spectinomycin resistance were located on two ribosomal RNA operons at different loci, indicating the pivotal role of ribosomal RNAs in conferring spectinomycin resistance in *L. enzymogenes*. The other mutants harbored mutations in the *tuf*, *rpoD*, *mltB*, and *purB* genes. Among them, the *tuf* and *rpoD* genes, respectively, encode a translation elongation factor Tu and an RNA polymerase primary sigma factor. They both contribute to protein biosynthesis, where ribosomal RNAs play essential roles. The *mltB* gene, whose product is involved in cell-wall recycling, was not only associated with resistance against spectinomycin, but also conferred resistance to osmotic stress and ampicillin. In addition, mutation of the *purB* gene, for which its product is involved in the biosynthesis of inosine and adenosine monophosphates, led to decreased spectinomycin resistance. Addition of exogenous adenine at lower concentration in medium restored the growth deficiency in the *purB* mutant and increased bacterial resistance to spectinomycin. These results suggest that while cell-wall recycling and purine biosynthesis might contribute to spectinomycin resistance, target rRNAs play critical role in spectinomycin resistance in *L. enzymogenes*.

## Introduction

Spectinomycin, an aminocyclitol antibiotic originally derived from *Streptomyces spectabilis*, is active against a variety of gram-positive and gram-negative bacteria (Davies et al., 1965; Hyun et al., 2000; Scarff et al., 2019). Spectinomycin inhibits bacterial protein synthesis by binding to helix 34 of 16S rRNA in the 30S ribosomal subunits and interfering with tRNA translocation by the elongation factor G (EF-G; Hancock, 1981; Unemo et al., 2013). Under the tradename Trobicin, spectinomycin remains an effective option for the treatment of gonorrhea caused by *Neisseria gonorrhoeae*, one of the most common sexually transmitted bacterial infections (Unemo et al., 2013; Nabu et al., 2014). However, resistance to spectinomycin was subsequently emerged and reported (Boslego et al., 1987; O’connor and Dahlberg, 2002). In *N. gonorrhoeae*, a single C1192U conversion in helix 34 on 16S rRNA resulted in high level of resistance to spectinomycin with a minimum inhibitory concentration (MIC) over 1,024 μg/ml (Galimand et al., 2000; Unemo et al., 2009). Furthermore, a deletion of codon 27 (valine) and a K28E mutation in 30S ribosomal protein S5 led to similarly high levels of spectinomycin resistance in *N. gonorrhoeae* (MIC > 1,024 μg/ml; Unemo et al., 2013). In other bacterial species, enzymatical modification of spectinomycin by adenylylation also conferred antibiotic resistance. Spectinomycin-resistant bacteria produced adenyltransferases to inactivate the drug and these enzymes are encoded by the *aad9*, *spd*, and *aadA14* genes, in *Enterococcus faecalis*, *Staphylococcus aureus*, and *Pasteurella multocida*, respectively (LeBlanc et al., 1991; Kehrenberg et al., 2005; Jamrozy et al., 2014).

Antibiotic resistance is currently a serious global threat to human, agriculture, and environment health due to the emerging, spread, and persistence of antibiotic-resistant and multidrug-resistant (MDR) bacteria (Aslam et al., 2018). As an example, delay of disease control caused by failure of antibiotic application could lead to significantly higher rates of hospital mortality (Cosgrove et al., 2003; Eliopoulos et al., 2003; Patel et al., 2008; Borer et al., 2009; Friedman et al., 2016). Over 2 million people in United States develop serious infectious diseases caused by bacterial pathogens resistant to antibiotics each year, and as a result, more than 23,000 of them die (Centers for Disease Control and Prevention, 2018). In agriculture, streptomycin was introduced into chemical control of bacterial diseases in the 1950s and proved to be one of the most effective bactericides for treatment of plant diseases caused by *Xanthomonas* spp., *Erwinia amylovora*, and *Pseudomonas* spp. (McManus et al., 2002). However, streptomycin control has been impeded by resistance evolved in plant pathogen populations (Cooksey, 1990). It is estimated that 16,465 kg antibiotics was applied to orchards in the United States in 2009 (Stockwell and Duffy, 2012). Moreover, contemporary animal husbandry relies heavily on antimicrobials used as therapeutics, prophylactics, and/or growth promotors (Woolhouse et al., 2015). It is estimated that food-animal production purchases approximately 80% of total antibiotics annually used in the United States (US Food and Drug Administration, 2014). Furthermore, worldwide growth in aquaculture is also accompanied by swift increase in the use of antibiotics. Similar with what happened in other agricultural field, about 80% of aquaculture-used antimicrobials enter environment in their intact forms and serve as a selector for environmental bacteria with antibiotic resistance genes (Cabello et al., 2013).

Comprehensively understanding the underlying molecular mechanisms of antibiotic resistance could help fight the growing threat of antibiotic-resistant bacteria. Previous studies have revealed that bacteria could develop antibiotic resistance during the process of antibiotic entry, accumulation, target binding, and toxicity. Genetic changes, including point mutations, recruitment of preexisting elements, and/or horizontal gene transfer (HGT), contribute to resistant development (Yelin and Kishony, 2018). Spatial exclusion of antibiotics into the cell by reduced permeability (Kato et al., 2003; Tran et al., 2013) or increased efflux (Wilson et al., 2011), and enzyme modification of drugs are among the most commonly discovered. As an example, β-lactamases cleave typical β-lactam rings and inactivate β-lactam antibiotics (Wilson et al., 2011; Yu and Zhao, 2020). Phosphotransferases, nucleotidyltransferases, and acetyltransferases modify aminoglycoside antibiotics (kanamycin, gentamycin, and streptomycin) by adding phosphoryl, adenylyl, or acetyl groups, respectively (Norris and Serpersu, 2013). In addition, target modifications, such as amino acid changes in β subunit of bacterial RNA polymerase, result in resistance to rifampicin (Goldstein, 2014). Streptomycin resistance could be induced by mutations in the *rpsL* (encoding the ribosomal S12 protein) or *rrs* gene (encoding 16S rRNA helices; Nair et al., 1993; Patel et al., 2017; Dal Molin et al., 2018). However, little is known about the antibiotic-resistant mechanisms in environmental bacteria.

*Lysobacter* species are ubiquitous in diverse environments, e.g., soil, rhizosphere, and freshwater (Christensen and Cook, 1978; Hayward et al., 2010). Several *Lysobacter* strains were found to inhibit multiple plant pathogens, thus considered as promising biocontrol agents (Giesler and Yuen, 1998; Kato et al., 1998; Chen et al., 2006; Qian et al., 2014; Yu et al., 2020). Further studies revealed that the bacterium produced a series of natural products with antimicrobial activities, including heat-stable antifungal factor (HSAF; Lou et al., 2010), alteramide B (ATB; Tang et al., 2019), WAP-8294A (Zhang et al., 2011), lysobactin (O'Sullivan et al., 1988), tripropeptins (Hashizume et al., 2001), and cephabacins (Panthee et al., 2016), making *Lysobacter* a potential resource for novel antibiotics. Another important characteristic of *Lysobacter* species is its intrinsic resistance to multiple antibiotics (Zhang et al., 2017; Yu and Zhao, 2019). Previous studies showed that *Lysobacter* resistomes were more abundant than that of *Xanthomonas campestris*, a plant pathogen in the *Xanthomonadaceae* family, and that *Lysobacter* species exhibited high level of multidrug resistance, especially to ampicillin and spectinomycin (Yu and Zhao, 2019). In *Lysobacter enzymogenes*, the most studied *Lysobacter* species; the inter-kingdom signal indole decreases its resistance against ampicillin and kanamycin by activating a dual importer that could transfer both vitamin B_12_ and antibiotics into bacterial cells (Wang et al., 2019). Moreover, cell permeability, β -lactamase activity, and transport were involved in the high level of resistance to ampicillin in *L. enzymogenes* (Yu and Zhao, 2020). The purpose of this study was to identify and functionally characterize genes associated with the elevated level of spectinomycin resistance in *L. enzymogenes*.

## Materials and methods

### Bacterial strains, plasmids, and culture conditions

The bacterial strains and plasmids used in this study are listed in Table 1. *Lysobacter* strains were routinely cultured in 14-ml round bottom Falcon® tubes (Corning Life Sciences) at 28°C with shaking at 250 rpm. Luria-Bertani (LB, Invitrogen, Carlsbad, CA, United States) broth was used for overnight growth and 10% tryptic soy medium (10% TSB, 1.5 g/L tryptone, 0.5 g/L soytone, and 0.5 g/L NaCl) was used for determination of antibiotic resistance. For *Escherichia coli* strains, LB was used for routine growth at 37°C. Antibiotics were used at the following concentrations unless otherwise noted: 100 μg/ml kanamycin (Km), 30 μg/ml gentamicin (Gm), and 10 μg/ml trimethoprim (Tmp). Primers used in this study for inverse/random amplification of transposon ends (RATE) PCR, mutant confirmation, cloning, and sequencing were listed in Supplementary Table S1.

**Table 1:** Bacterial strains and plasmids used in this study.

**Strains, plasmids**	**Description**	**Reference or source**
**Strains**		
*Lysobacter enzymogenes* C3 (SAMN03275730)	LeC3, wild-type, isolated from Kentucky bluegrass foliage, Nebraska, Km^R^	Giesler and Yuen, 1998
*Lysobacter antibioticus* ATCC29479	LaATCC29479, wild-type, isolated from the soil of Central Experimental Farm, Ontario, Canada	Christensen and Cook, 1978
*Escherichia coli* DH10B	F– *mcr*A ∆(*mrr-hsd*RMS*-mcr*BC) Ф80*lacZ*∆M15 ∆*lac*X74 *rec*A1 *end*A1 *ara*D139 ∆ (*ara leu*) 7697 *gal*U *gal*K *rps*L *nup*G λ–	Invitrogen (Carlsbad, CA, United States)
*16S rRNA*	Transposon insertion mutant in gene *GLE_6063* (encoding one 16S ribosomal RNA) at 965,406, Km^R^	This study
*23S rRNA*	Transposon insertion mutant in gene *GLE_6061* (encoding one 23S ribosomal RNA) at 292,133, Km^R^	This study
*rpoD*	Transposon insertion mutant in gene *GLE_0368* (encoding one RNA polymerase primary sigma factor) at 408,359, Km^R^	This study
*mltB*	Transposon insertion mutant in gene *GLE_4483* (encoding one lytic murein transglycosylase B) at 5,029,914, Km^R^	This study
*purB*	Transposon insertion mutant in gene *GLE_2716* (encoding one adenylosuccinate lyase) at 3,056,168, Km^R^	This study
*E. coli* MY101	Derivative of SQ171 (*∆7 prrn* strain), a null mutant of the rRNA (*rrn*) operons in the chromosome. *∆rrnG ∆rrnA ∆rrnD ∆rrnE ∆rrnH ∆rrnB ∆rrnC*	Miyazaki and Kitahara, 2018
**Plasmids**		
pBBR1MCS-5	Broad-host-range vector with a Plac promoter, Gm^R^	Kovach et al., 1995
pMltB	1717-bp DNA fragment containing the promoter and coding sequence of *mltB* (*GLE_4483*) (−376 to +1,341) of LeC3 in pBBR1MCS-5, Gm^R^	This study
pPurB	1955-bp DNA fragment containing the promoter and coding sequence of *lablaL2* of and one hypothetical sequence *purB* (*GLE_2716*) (−442 to +136) of LeC3 in pBBR1MCS-5, Gm^R^	This study
pMY101	Constructed by transferring the tRNA gene cluster encoded by pTRNA67 into pRB101 at the site between the 16S and 23S rRNA genes. Resulted plasmid contains the entire *E. coli rrnB* operon (*E. coli rrnB*, tRNA^Glu^, tRNA^Asp.,^ tRNA^Ile^, tRNA^Ala^, tRNA^Trp^, *sacB*, Amp^R^, pSC101 ori).	Miyazaki and Kitahara, 2018
pMY205mPAG2	Used as a vector to introduce foreign 16S rRNA genes. *E. coli* rrnB, tRNA^Glu^, tRNA^Asp.,^ tRNA^Ile^, tRNA^Ala^, tRNA^Trp^, Tmp^R^, p15a ori	Miyazaki and Kitahara, 2018
pMY-Ec	16S rRNA on *rrnB* operon replaced by wild-type 16S rRNA gene of *E. coli* DH10B in pMY205mPAG2, Tmp^R^	This study
pMY-LeC3	16S rRNA on *rrnB* operon replaced by wild-type 16S rRNA gene of *L. enzymogenes* C3 in pMY205mPAG2, Tmp^R^	This study
pMY-LaATCC	16S rRNA on *rrnB* operon replaced by wild-type 16S rRNA gene of *L. antibioticus* ATCC29479 in pMY205mPAG2, Tmp^R^	This study

LeC3, *Lysobacter enzymogenes* strain C3; Gene bank accession no. CP013140 (www.ncbi.nlm.nih.gov/nuccore/951301122).

### Transposon mutagenesis, screening, determination of insertion sites, and complementation of the selected mutants

The EZ-Tn5™ < KAN-2 > Tnp Transposome™ kit was used for random mutagenesis following the manufacturer’s instructions (Epicenter, Madison, WI, United States). Briefly, 1 μl of the transposome was electroporated into the wild-type LeC3. Transformants were plated and selected from LB amended with 2,000 μg/ml Km onto both LB amended with 2,000 μ g/ml Km (Plate A) and LB amended with 2,000 μ g/ml Km plus 1,000 μ g/ml spectinomycin (Spc, Plate B). Colonies grown on Plate A, but not on Plate B, were selected for second screening in 96-well plate. After 22 h of incubation, bacterial growth (OD_600_) was measured in 10% TSB amended with 0, 100, 200, and 400 μg/ml Spc. Since 400 μg/ml Spc has no influence on LeC3, mutants with obviously reduced growth in 400 μg/ml Spc were selected for determination of transposon insertion sites using RATE or inverse PCR as described previously (Yu and Zhao, 2020). PCR products were then gel-purified and sequenced at the University of Illinois at Urbana-Champaign core sequencing facility. Flanking sequences of the transposon insertion sites were searched using BLAST against LeC3 genome at NCBI. Different primer pairs were used to confirm the identified insertion sites (Supplementary Table S1). For complementation of mutants, the DNA fragments containing the native promoter and coding sequence of the selected genes were amplified by PCR using primer pairs for the genes (Supplementary Table S1) and cloned into pBBR1MCS-5 (Kovach et al., 1995; Yu and Zhao, 2019). The resulting plasmids were verified by sequencing and electroporated into the corresponding mutants.

### Bacterial growth in 10% TSB

Bacterial growth was measured as previously described (Lee and Zhao, 2015). Briefly, overnight cultures of bacterial strains were harvested by centrifugation at 4,000-rpm for 10 min and washed twice using 0.5 × phosphate-buffered saline (0.5 × PBS). After the final wash, the pellet was resuspended in 10% TSB and adjusted to OD_600_ = 0.02. Bacterial strains were cultured at 28°C with 250-rpm shaking. Aliquots of cultures were taken and OD_600_ was measured to determine bacterial growth at different time points. The experiments were repeated three times.

### Antibiotic/adenine test

*Lysobacter enzymogenes* strain C3 and its derived strains were grown overnight, harvested by centrifugation, and washed twice using 0.5 × PBS. After the final wash, cells were resuspended in 10% TSB medium and 10% TSB amended with antibiotics and/or adenine (adenine hemisulfate salt, Sigma-Aldrich, St. Louis, Missouri, United States). The initial concentration of bacterial suspension was adjusted to OD_600_ = 0.02. After incubation at 28°C with shaking at 250 rpm for 22–24 h, bacterial growth (OD_600_) was measured. MIC_50_ was defined as the concentration range of antibiotic at which bacterial growth was <50% of that of no-antibiotic control. In contrast, the initial concentration for suspension of overnight *E. coli* derived strains was OD_600_ 0.05 in LB broth amended with Tmp or Tmp/Spc. After incubation at 37°C with shaking at 250 rpm for 15 h, OD_600_ was measured. For both LeC3- and *E. coli*-derived strains, relative growth was calculated as the ratio of bacterial OD_600_ with Spc to that without Spc and was used to represent resistance to Spc. The experiments were repeated three times.

### Expression of exogenous 16S rRNA genes into null-*rrn* (ribosomal RNA) *Escherichia coli* strain MY101

The 16S rRNA gene fragments were PCR-amplified from *E. coli* strain DH10B, *L. enzymogenes* strain C3 (LeC3), and *L. antibioticus* strain ATCC29479 (LaATCC) using primer pairs (Supplementary Table S1) and cloned to replace the *E. coli* 16S rRNA gene in pMY205mPAG2 with Tmp^R^ (Miyazaki and Kitahara, 2018). The resulting plasmids were verified by sequencing and electroporated into the null-*rrn E. coli* strain MY101 with pMY101 (Amp^R^, resistance to ampicillin; *sacB*, susceptible to sucrose). Replacement of pMY101 by pMY205mPAG2-derived vectors was conducted as described previously (Miyazaki and Kitahara, 2018). Briefly, transformants after electroporation were spread on LB/Tmp agar plates. Recovered colonies with pMY205mPAG2-derived vector were resuspended in LB broth and spread on LB/Tmp agar containing 5% (w/v) sucrose (Plate C) to eliminate pMY101. The recovered colonies were selected onto both Plate C and LB agar containing 100 μg/ml ampicillin (Plate D). Colonies grown on Plate C, but not on Plate D, were *E. coli* strains MY101 with exogenous 16S rRNA genes successfully introduced, which were then verified using PCR.

### Spot dilution assay

Spot dilution assay was performed using a previously described procedure (Ge et al., 2018; Yu and Zhao, 2020). Briefly, overnight bacterial cells were harvested by centrifugation and washed twice using 0.5 × PBS. After the final wash, the pellet was resuspended in 0.5 × PBS and adjusted to OD_600_ = 1.0. Tenfold serial dilutions of the bacterial suspension were made in 0.5 × PBS. Each dilution (5 μl) was spotted on the plates with different concentrations of NaCl (8.85 or 250 mM) and incubated at 28°C for 3 days. The experiment was performed in duplicate and repeated three times.

### Nitrocefin assay

*Lysobacter enzymogenes* strain C3 and its derived strains were tested for their abilities to cleave the chromogenic β -lactamase substrate nitrocefin as previously described (Yu and Zhao, 2020). One-day bacterial cultures were centrifuged and resuspended to OD_600_ = 1.5 in 0.5 × PBS. Bacterial suspensions (75 μl) were transferred to duplicate wells in a 96-well plate and control wells were seeded with 0.5 × PBS only. To each well, 75 μl of a 250 μg/ml nitrocefin (Calbiochem®) solution in 0.5 × PBS was added and the absorbance at a wavelength of 486 nm (A_486_) was measured using a microplate spectrophotometer (SpectraMax®) after 0 and 2 h of incubation at room temperature. The experiment was performed in duplicate and repeated three times.

### Statistical analysis

Bacterial growth and resistance data were compared using a one-way ANOVA followed by Fisher’s Least Significant Difference (LSD) test to determine differences in means (*p* = 0.05) using the SAS 9.4 (Cary, NC, United States). Changes marked with the same letter did not differ significantly (*p* < 0.05).

## Results

### Nineteen mutants were recovered with decreased spectinomycin resistance

**Figure 1:**
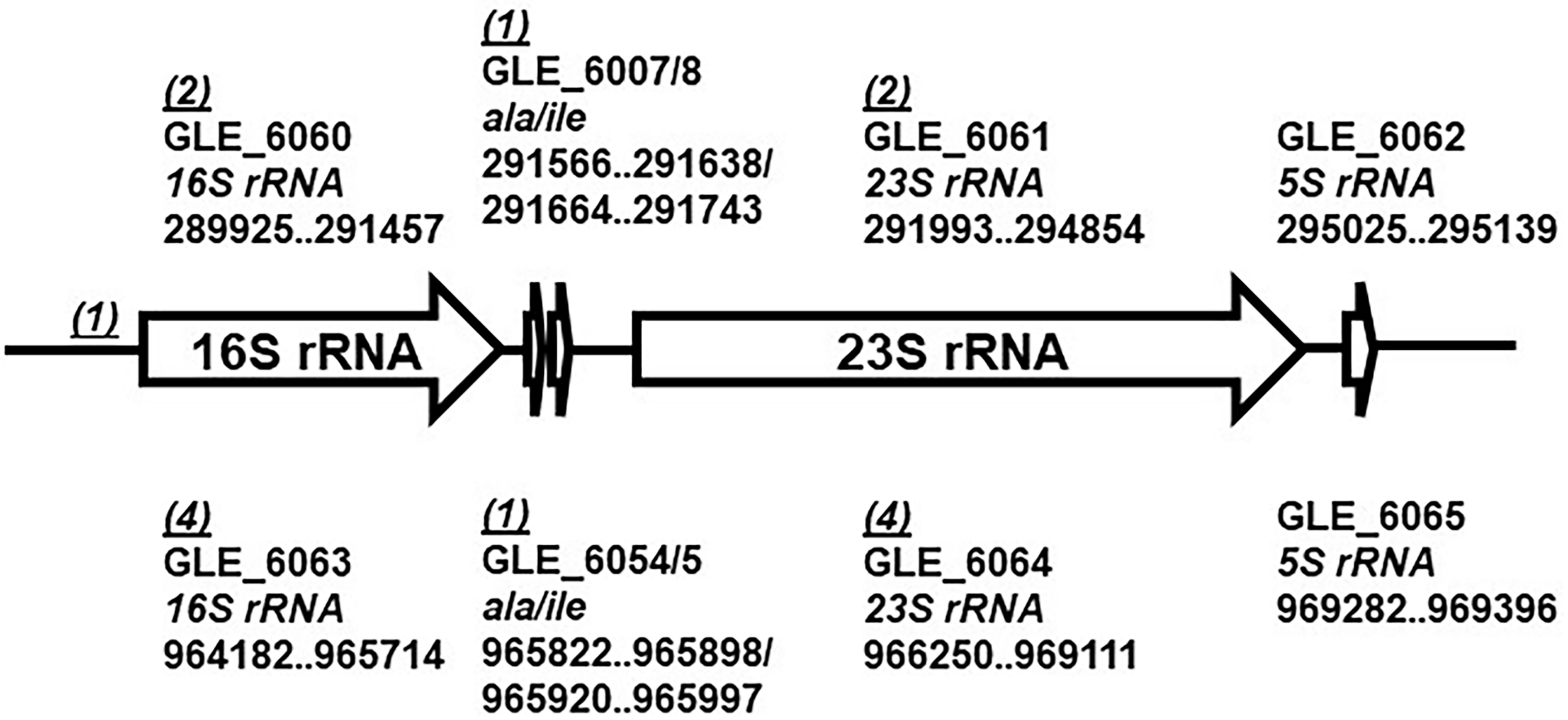
Schematic map of the two rRNA operons in *Lysobacter enzymogenes*. Each ribosomal RNA (*rrn*) operon contains five genes, encoding 16S rRNA (*16S rRNA*), tRNA-Ala (*ala*), tRNA-Ile (*ile*), 23S rRNA (*23S rRNA*), and 5S rRNA (*5S rRNA*). Numbers in parentheses indicate numbers of mutants identified with insertions in the gene or the intergenic region (IGR). The genome sequence accession number for *L. enzymogenes* C3 is CP013140.

In our previous study, we reported that LeC3 exhibited a high level of resistance to spectinomycin (Yu and Zhao, 2019). In order to illustrate the underlying molecular mechanisms, a mutant library of 6,985 clones was screened and 19 mutants sensitive to spectinomycin were recovered (Supplementary Table S2). The insertion sites of these 19 mutants were then determined by either RATE PCR or inverse PCR followed by sequence alignment against the complete genome of LeC3 (gene bank accession no. CP013140). Among them, 15 mutants were located on two ribosomal RNA operons at different loci, with six mutants each in the *16S* and *23S rRNA* genes at different sites, two mutants in the *ala* gene at different sites, and one mutant in the intergenic region (IGR) between the *tyrS* gene encoding a tyrosyl-tRNA synthetase and the *16S* rRNA (Figure 1). Other mutations were in genes encoding a translation elongation factor Tu (*tuf*), an RNA polymerase primary sigma factor (*rpoD*), a lytic murein transglycosylase B (*mltB*), and an adenylosuccinate lyase (*purB*).

**Figure 2:**
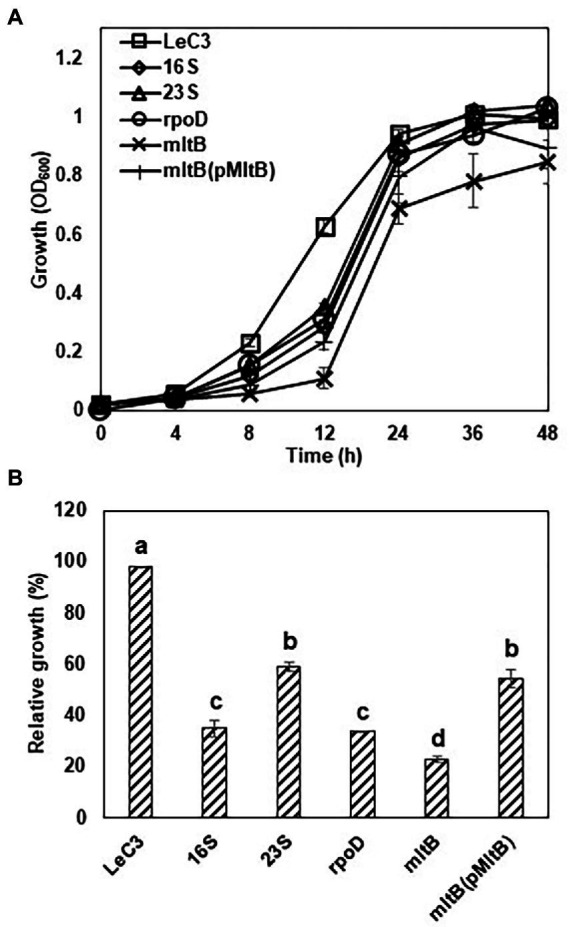
Selected mutants exhibited decreased spectinomycin resistance and slower growth. **(A)** Growth curve of *Lysobacter enzymogenes* strain C3 (LeC3) and its derived strains. Bacterial growth was monitored at different time points by measuring OD_600_. Data points represented the means of three replicates ± SD. **(B)** Spectinomycin resistance of LeC3 and its derived strains. Spectinomycin resistance was calculated as the ratio of bacterial growth with 400 μg/ml spectinomycin to bacterial growth without spectinomycin at 22 h post-inoculation. Error bars represented SD. Different letters indicated significant differences from one-way ANOVA followed by Fisher’s LSD test (*p* < 0.05). LeC3, *Lysobacter enzymogenes* strain C3; *16S*, the *16S rRNA* mutant; *23S*, the *23S rRNA* mutant; *rpoD*, the *rpoD* mutant; *mltB*, the *mltB* mutant; and LaATCC, *L. antibioticus* strain ATCC29479.

To further determine spectinomycin susceptibility for the mutants, the growth of four mutants, including one each of the *16S rRNA*, *23S rRNA*, *rpoD*, and *mltB* mutants, was determined in 10% TSB. The *16S rRNA*, *23S rRNA*, and *rpoD* mutants grew slower in early hours than that of LeC3, but reached similar level as LeC3 at 24 h post-inoculation (hpi); whereas the *mltB* mutant exhibited about 30% decreased growth at 24 hpi (Figure 2A). Due to delayed growth, relative growth was calculated to reflect spectinomycin susceptibility and normalized to OD_600_ of 1. Results showed that the four selected mutants exhibited different levels of spectinomycin susceptibility as compared with LeC3 (Figure 2B). The *mltB* mutant was highly susceptible to spectinomycin and the cloned *mltB* gene partially complemented the *mltB* mutant in both spectinomycin susceptibility and growth (Figures 2A,B). However, there was no significant difference between the spectinomycin susceptibility of the *16S* and *rpoD* mutants.

In addition, the minimum inhibitory concentration 50 (MIC_50_) for the *16S rRNA*, *23S rRNA*, *rpoD*, and *mltB* mutants was also determined. As compared with LeC3 with an MIC_50_ at 1,500–2,000 μ g/ml, the MIC_50_ for the selected mutants (*16S rRNA*, *23S rRNA*, *rpoD*, and *mltB*) were 200–300, 800–1,000, 100–200, and <100 μ g/ml, respectively (Table 2). MIC_50_ for the complementation strain of the *mltB* mutant was partially restored (200–500 μ g/ml, Table 2).

**Table 2:** Minimum inhibition concentration 50 (MIC_50_) of LeC3 and its derived strains for spectinomycin and ampicillin.

**Strains**	**Spc MIC**_**50**_ **(μg/ml)**	**Amp MIC**_**50**_ **(μg/ml)**
LeC3	1,500–2,000	>500
*16S rRNA*	200–300	N/D
*23S rRNA*	800–1,000	N/D
*rpoD*	100–200	N/D
*mltB*	0–100	100–200
*mltB* (pMltB)	200–500	>500
LeC3 (0.5 mM adenine)	>2,500	N/D

MIC_50_ was defined as the concentration range of spectinomycin or ampicillin in which growth (OD_600_) of the bacterium was <50% of that of the control without antibiotic. N/D, not determined; Spc, spectinomycin; Amp, ampicillin; LeC3, *Lysobacter enzymogenes* strain C3; 16S rRNA, the 16S rRNA mutant; 23S rRNA, the 23S rRNA mutant; rpoD, the rpoD mutant; and *mltB*, the *mltB* mutant.

### The 16S rRNA from LeC3 conferred resistance to spectinomycin in null-*rrn Escherichia coli* strain MY101

*Lysobacter enzymogenes* contains two identical ribosomal RNA (*rrn*) operons, each with five genes, encoding 16S rRNA (*16S rRNA*), tRNA-Ala (*ala*), tRNA-Ile (*ile*), 23S rRNA (*23S rRNA*), and 5S rRNA (*5S rRNA*; de Bruijn et al., 2015; Figure 1). Among the 19 mutants with decreased spectinomycin resistance in LeC3 (Supplementary Table S2), five and nine mutants harbored insertions in the *rrn* operon 1 (289,925–29,513) and 2 (964,182–969,396), respectively (Figure 1). These results suggested that mutations of either *16S rRNA*, *23S rRNA*, or *ala* genes on either of the two *rrn* operons in LeC3 led to increased spectinomycin susceptibility, indicating that the *rrn* operons act as the main targets conferring resistance to spectinomycin in LeC3.

**Figure 3:**
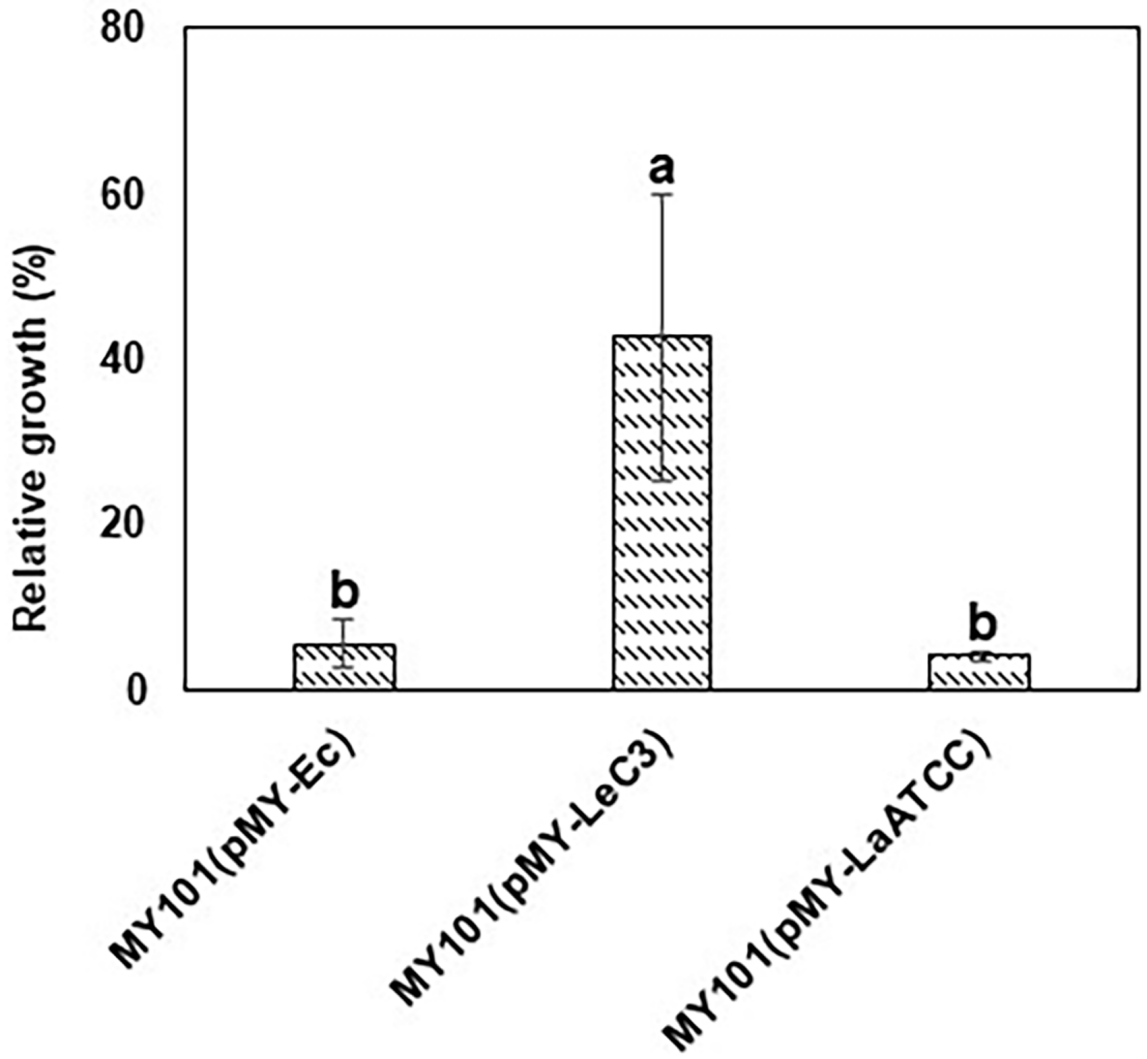
16S rRNA from LeC3 conferred resistance to spectinomycin (Spc) as compared with 16S rRNA from LaATCC29479 in *Escherichia coli* strain MY101. Relative growth was calculated as the ratio of growth (OD_600_) with 64 μ g/ml Spc to bacterial growth without Spc 24 h after inoculation. Error bars represented SD. Different letters indicated significant differences from one-way ANOVA followed by Fisher’s LSD test (*p* < 0.05).

Since spectinomycin binds 16S rRNA component of the 30S subunits, we utilized an *E. coli* null mutant of the *rrn* operons in the genome to examine whether 16S rRNA from LeC3 confers resistance to spectinomycin in *E. coli*. Previous studies also showed that LeC3 displayed higher level of resistance to spectinomycin as compared with that of *L. antibioticus* ATCC29479 (LaATCC29479; Yu and Zhao, 2019). Therefore, we also compared wild-type 16S rRNA from LeC3 with that of LaATCC29479. First, full-length *16S rRNA* genes individually replaced an *E. coli 16S rRNA* gene in pMY205mPAG2, which originally contained a complete *E. coli rrnB* operon. The resulting plasmids were then individually introduced into an *E. coli* null mutant of the *rrn* operons in the genome (strain MY101; Miyazaki and Kitahara, 2018). Results showed that MY101 strain harboring the 16S rRNA from LeC3 exhibited relative faster growth as compared with those containing the 16S rRNA from *E. coli* or LaATCC29479 with spectinomycin resistance at 64 μ g/ml (Figure 3), suggesting that 16S rRNA of LeC3, but not LaATCC29479, conferred resistance to spectinomycin in null-*rrn E. coli* strain MY101.

### Besides spectinomycin, the *mltB* mutant was susceptible to osmotic stress and ampicillin

**Figure 4:**
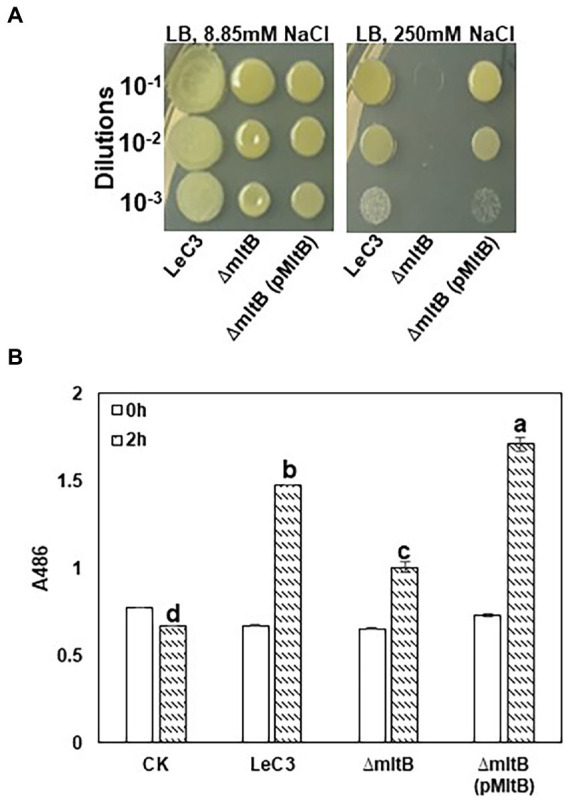
The *mltB* gene controls osmotic stress response and β -lactamase activity. **(A)** The *mltB* mutant showed decreased resistance to osmotic stress. Serial 10-fold dilutions were made from OD_600_ = 0.1 in 0.5 × PBS. Each dilution was added to LB plates containing 8.85 mM NaCl (left panel) and 250 mM NaCl (right panel). Photographs were taken 3 days post-inoculation. **(B)** Quantitative measurements of β -lactamase activity in LeC3 and its derived strains. The β -lactamase activity was measured at 486 nm (A_486_) using the chromogenic compound nitrocefin at 2 h post-inoculation. Error bars represented SD. Different letters indicated significant differences from one-way ANOVA followed by Fisher’s LSD test (*p* < 0.05). LeC3, *Lysobacter enzymogenes* strain C3; *mltB*, the *mltB* mutant; and CK, 0.5 × phosphate-buffered saline.

As the *mltB* gene encodes one of the major cell-wall recycling enzymes, i.e., the lytic murein transglycosylase B (MltB), we hypothesized that growth deficiency and decreased resistance to spectinomycin in the *mltB* mutant might be associated with its increased susceptibility to osmotic shock. Spot dilution assay revealed that the *mltB* mutant showed similar growth with LeC3 on LB plates with lower concentration of NaCl (8.85 mM). The *mltB* mutant was more susceptible to osmotic stress (three dilutions lower on LB plates containing 250 mM NaCl) as compared with LeC3 and the complementation strain was partially restored (Figure 4A). These results suggest that the *mltB* gene might be involved in osmotic response in *L. enzymogenes*.

Ampicillin is an antibiotic that targets cell-wall biosynthesis and previous report showed that LeC3 exhibited a high level of resistance to ampicillin (Yu and Zhao, 2019, 2020). To explore whether the *mltB* gene is also involved in ampicillin resistance, MIC_50_ of LeC3, the *mltB* mutant, and its complementation strain was determined. As compared with LeC3 with an MIC_50_ > 500 μ g/ml, the MIC_50_ for the *mltB* mutant was 100–200 μ g/ml and the mutant was partially complemented (Table 2). The β -lactamase activity in the *mltB* mutant was significantly lower as compared with that of LeC3 and the complementation strain (Figure 4B), suggesting that the difference of LeC3 and the *mltB* mutant in ampicillin resistance might be partially due to their difference in β -lactamase activities.

### Purine biosynthesis contributed to resistance to spectinomycin in LeC3

**Figure 5:**
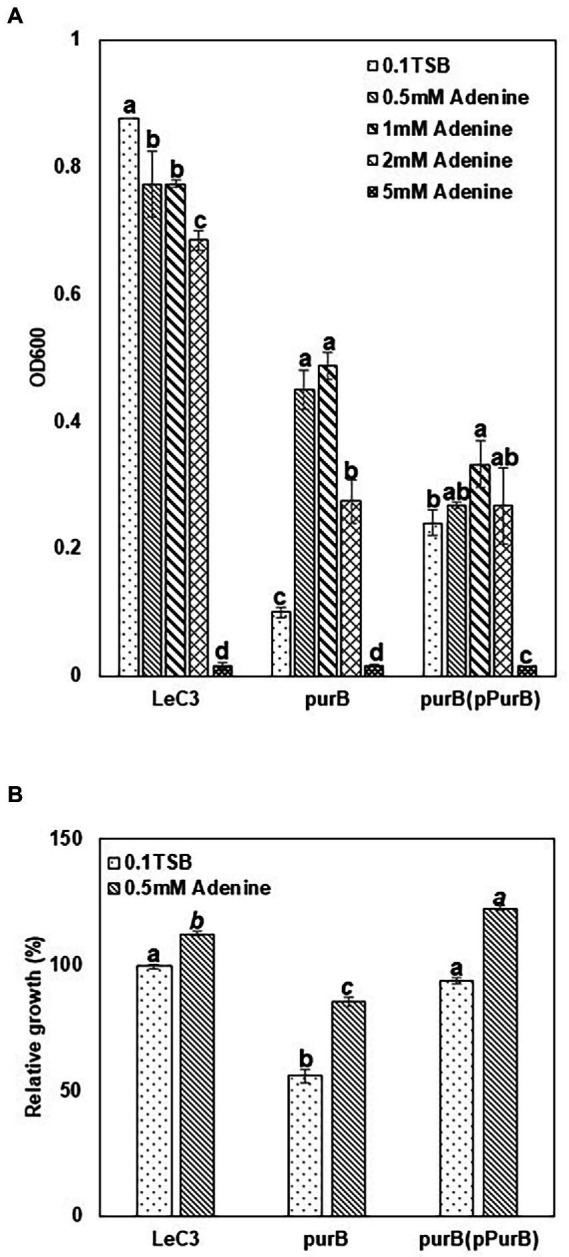
Purine biosynthesis contributed to resistance to spectinomycin in LeC3. **(A)** Growth of LeC3 and its derived strains on different adenine concentration at 24 hpi. The absorbance at 600 nm was measured to represent bacterial growth. Within each strain, different letters indicated significant differences among different concentrations of adenine from one-way ANOVA followed by Fisher’s LSD test (*p* < 0.05). In addition, “ab” means significant difference with “c,” but not with “a” or “b.” **(B)** Spectinomycin resistance of LeC3 and its derived strains with and without adenine at 24 hpi. Spectinomycin resistance was calculated as the ratio of bacterial growth with 500 μg/ml spectinomycin to bacterial growth without spectinomycin. Within each concentration of adenine, different letters indicated significant differences among different bacterial strains from one-way ANOVA followed by Fisher’s LSD test (*p* < 0.05). Error bars represented SD. LeC3, *Lysobacter enzymogenes* strain C3; *purB*, the *purB* mutant.

One recovered mutant (Supplementary Table S2) harbored a mutation in the *purB* gene, which encodes an adenylosuccinate lyase and is involved in purine biosynthesis. However, severe deficiency in growth was observed in the *purB* mutant in 10% TSB at 24 hpi as compared with LeC3 (Figure 5A). This severe deficiency of growth in the *purB* mutant was partially restored by either addition of 0.5–1 mM adenine to the medium or introduction of the *purB* gene (Figure 5A). No significant growth difference was observed for the complementation strain in 10% TSB or by adding 0.5–2 mM adenine, but not 5 mM adenine, which inhibited its growth (Figure 5A). In addition, higher concentration of adenine (2 or 5 mM) also led to inhibition of bacterial growth in both LeC3 and the mutant (Figure 5A). Therefore, 0.5 mM adenine was used for spectinomycin susceptibility tests. Results showed that with and without 0.5 mM adenine, the *purB* mutant exhibited significantly lower level of resistance to spectinomycin as compared with LeC3 and the complementation strain (Figure 5B). Interestingly, addition of 0.5 mM adenine to the medium increased bacterial resistance to spectinomycin in all three strains tested (Figure 5B; Table 2), further suggesting that purine biosynthesis might contribute to spectinomycin resistance in LeC3 as well.

## Discussion

There is a critical need in comprehensively understanding the underlying molecular mechanisms of antibiotic resistance in environmental microorganisms. *L. enzymogenes*, with an elevated level of intrinsic MDR, is an appropriate surrogate for studies in antibiotic resistance and in understanding how to circumvent the antibiotic crisis in clinical pathogens. We previously reported that *L. enzymogenes* exhibited a high level of resistance to spectinomycin (Yu and Zhao, 2019). In this study, we identified 19 mutants in *L. enzymogenes* with decreased resistance to spectinomycin and demonstrated that spectinomycin susceptibility in these mutants is mostly due to changes of its ribosomal RNA targets, and also related to cell-wall recycling and purine biosynthesis.

Bacteriostatic spectinomycin interferes with bacterial growth *via* binding to the 30S ribosomal subunits to inhibit protein synthesis (Hancock, 1981). Among the 19 mutants with decreased spectinomycin resistance, 16 were involved in bacterial protein synthesis, including six each of 16S rRNA or 23S rRNA, two alanine tRNA, and the *tuf* gene, encoding a translation elongation factor Tu, which promotes the GTP-dependent binding of aminoacyl-tRNA to the A-site of ribosomes during protein biosynthesis (Daviter et al., 2003). Previous proteomic analysis of a *N. gonorrhoeae* strain with high resistance to spectinomycin revealed that the *tuf* gene was highly expressed in the resistant strain. It is possible that high expression of Tuf might balance and maintain protein biosynthesis in response to inhibition by spectinomycin in the resistant strain (Nabu et al., 2014). This might also be true in *L. enzymogenes*, which needs further investigation. On the other hand, RpoD, as the house-keeping sigma factor, is required in the initiation step of transcription by specifically recognizing promoter sequences of bacterial essential genes, i.e., ribosomal genes and protein synthesis (Nagai and Shimamoto, 1997; Davis et al., 2017). Therefore, the *rpoD* mutant exhibited spectinomycin susceptibility probably due to its indirect involvement in protein biosynthesis by affecting expression of ribosome genes.

**Figure 6:**
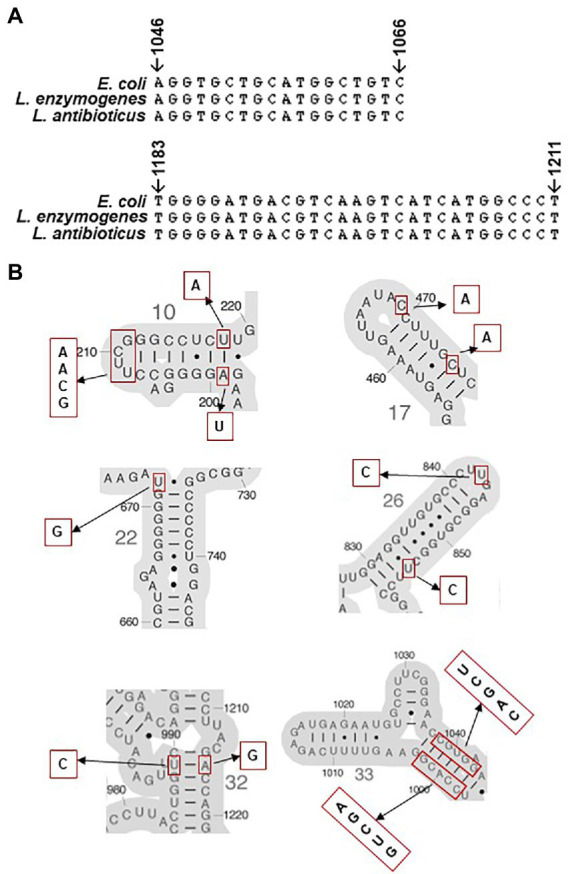
Comparison of 16S rRNA. **(A)** Multiple sequence alignment of hot spots in 16S rRNA genes from *Escherichia coli*, *Lysobacter enzymogenes*, and *Lysobacter antibioticus* with known spectinomycin resistance mutations. **(B)** Secondary structures of *E. coli* 16S rRNA around point mutation sites identified between *L. enzymogenes* and *L. antibioticus*. Putative resistance nucleotides to spectinomycin are marked using red rectangular and the corresponding sequences in LeC3 are shown on the side.

Previous studies revealed that the direct binding site of spectinomycin was at the helix 34 of *E. coli* 16S rRNAs between positions 1,046–1,065 and 1,191–1,211(Miyazaki and Kitahara, 2018). We searched mutations in 16S rRNA genes from organisms with altered spectinomycin resistance and found that hot spots where spectinomycin resistance mutations frequently discovered were around helix 34 and its neighboring positions (Supplementary Table S3). As an example, mutations C1066U and C1192U conferred spectinomycin resistance in *E. coli* (Sigmund et al., 1984; Johanson and Hughes, 1995). Additionally, mutations A1191G, C1192U, C1192G, and G1193C were associated with spectinomycin resistance in *Borrelia* spp. (Binet and Maurelli, 2005; Criswell et al., 2006). However, listing all point mutations associated with antibiotic resistance in rRNA (*rrn*) operons is challenging as multiple *rrn* operons normally exist in bacterial genomes, e.g., seven in *E. coli* and two in *L. enzymogenes*. Miyazaki and colleague developed a novel approach to circumvent this methodological problem by using a null-*rrn* mutant of *E. coli* as a test host to detect exogenous 16S rRNAs for altered spectinomycin resistance (Miyazaki and Kitahara, 2018). In this study, 16S rRNAs from *Lysobacter* species were expressed in null-*rrn* mutants of *E. coli* and results showed that 16S rRNA from LeC3 conferred spectinomycin resistance. However, sequence analysis of *16S rRNA* genes revealed no difference in the two hot spots (Figure 6A; Supplementary Table S3), where spectinomycin resistance mutations have already been discovered (Supplementary Table S3), indicating that unknown mutations associated with spectinomycin resistance might exist in the 16S rRNA of *L. enzymogenes*. Sequence alignment (Supplementary Figure S1) revealed that point mutations between the 16S rRNAs of LeC3 and LaATCC29479 were in six different regions, and these included helix 10 (*E. coli* numbering, 198–219, A199U, UUCG208-211GCAA, and U218A), helix 17 (455–477, C469A, and C475A), helix 22 (666–672 & 734–740, U672G), helix 26 (829–854, U843C, and U854C), helix 32 (984–990, 1,215–1,221, U989C, and A1216G), and helix 33 (996–1,046, CCACG998-1002GUCGA, and CGUGG1038-1043UCGAC), leading to different secondary structures (Figure 6B; Supplementary Figure S2). It is also possible that these mutations might be involved in their difference in conferring spectinomycin resistance, which requires further investigation. In addition, previous studies showed that the difference of LeC3 and LaATCC29479 in resistance to multiple antibiotics including spectinomycin was partly due to differences in cellular permeability (Yu and Zhao, 2020).

Cell walls are critical for the viability of the bacterium (Dik et al., 2018). Cell-wall remodeling is tightly regulated to guarantee bacterial survival and could also be directly associated with antibiotic resistance (Domínguez-Gil et al., 2016). In Gram-negative bacteria, cell-wall recycling starts with the lytic transglycosylases (LTs) by degrading peptidoglycan (PG), the major component of bacterial cell walls (Suvorov et al., 2008; Dik et al., 2018). Loss of LTs increased bacterial resistance to β -lactams in *Shewanella oneidensis*, but led to higher β -lactam susceptibility in *Pseudomonas aeruginosa* (Lamers et al., 2015; Yin et al., 2018). These findings suggest that PG metabolism could influence bacterial resistance. It has been reported that LT inactivation has the potential to reduce bacterial autolysis and increase survival time, thus antibiotic resistance (Lamers et al., 2015). On the other hand, inactivation of LTs could reduce the production of 1,6-anhydromuropeptides (anhMPs) from PG degradation, which binds to transcriptional activator AmpR, thus preventing the induction of β -lactamase expression (Jacobs et al., 1994; Mark et al., 2011). Decreased expression of β -lactamase consequently leads to β -lactam susceptibility. In this study, the *mltB* mutant in *L. enzymogenes* exhibited susceptibility to β -lactam ampicillin and showed decreased β -lactamase activity, suggesting that the *mltB* gene could influence PG metabolism and affect β -lactamase expression. Further studies are needed to confirm this hypothesis. In addition, AmpR is also associated with bacterial resistance to quinolones and aminoglycosides in *P. aeruginosa* by regulating MexEF-OprN efflux pump (Kumari et al., 2014). It is also reasonable to speculate that the *mltB* mutant in LeC3 exhibited ampicillin and spectinomycin susceptibility by possibly affecting AmpR activity in regulation of efflux pumps.

The adenylosuccinate lyase (ASL) encoded by the *purB* gene is involved in the *de novo* pathway of inosine monophosphate (IMP) and adenosine monophosphate (AMP) biosynthesis (Yang et al., 2021). Deletion of the *purB* gene resulted in severe growth deficiency in *L. enzymogenes*, probably caused by disrupted purine biosynthesis as reported previously in other bacteria (Jung et al., 2010; Faith et al., 2012). Consistent with the *purB* mutant of *Mesorhizobium loti*, 0.5 mM exogeneous adenine in medium partially restored its growth deficiency in the *purB* mutant of *L. enzymogenes* (Okazaki et al., 2007). We also found that higher concentration of adenine led to obvious inhibition of bacterial growth in *L. enzymogenes* and its derived strains. This bacteriostatic effect of adenine was consistent with what was previously described in *Aerobacter aerogenes* (Brooke and Magasanik, 1954; Moyed, 1964). Hosono and colleagues reported that adenine interfered bacterial growth by inhibiting *de novo* synthesis of pyrimidine nucleotides (Hosono and Kuno, 1974). The *purB* mutant with deficiency in purine biosynthesis exhibited decreased spectinomycin resistance. This is consistent with the genetic determination of oxacillin resistance in methicillin-resistant *Staphylococcus aureus* (MRSA), where the *purB* gene was identified as one of the genes linked to oxacillin resistance (Pardos de la Gandara et al., 2018). However, we found exogenous adenine could increase antibiotic resistance, a phenomenon not previously reported in bacteria. Further studies are needed to explain this phenomenon.

Taken together, our findings demonstrated that, ribosomal RNAs, the direct target of spectinomycin, play a pivotal role in conferring spectinomycin resistance in *L. enzymogenes*. Cell-wall recycling and purine biosynthesis might also play some roles in conferring spectinomycin resistance. However, further studies are needed to clarify which bases (or point mutations) in 16S rRNA lead to resistance to spectinomycin in LeC3. Further studies should also determine whether the mutation in the *mltB* gene resulted in decreased efflux pumps mediated by the transcriptional activator AmpR and how exogenous adenine or the *purB* mutation affects antibiotic resistance in LeC3.

## Data availability statement

The datasets presented in this study can be found in online repositories. The names of the repository/repositories and accession number(s) can be found in the article/Supplementary material.

## Author contributions

MY and YZ designed the research and wrote the manuscript. MY performed the research and analyzed the data. All authors contributed to the article and approved the submitted version.

## Funding

This project was supported by the Agriculture and Food Research Initiative Competitive grants Program grant no. 2016-67013-24812 from the USDA National Institute of Food and Agriculture, startup and endowed funds (YZ).

## Conflict of interest

The authors declare that the research was conducted in the absence of any commercial or financial relationships that could be construed as a potential conflict of interest.

## Publisher’s note

All claims expressed in this article are solely those of the authors and do not necessarily represent those of their affiliated organizations, or those of the publisher, the editors and the reviewers. Any product that may be evaluated in this article, or claim that may be made by its manufacturer, is not guaranteed or endorsed by the publisher.
