# Supplementary material for: Spectinomycin resistance in Lysobacter enzymogenes is due to its rRNA target but also relies on cell-wall recycling and purine biosynthesis
Source: Front Microbiol. 2022 Aug 31;13:988110. doi: 10.3389/fmicb.2022.988110 (PMC9471086; doi:10.3389/fmicb.2022.988110)
Supplement: Supplementary file 1 [file Data_Sheet_1.pdf]

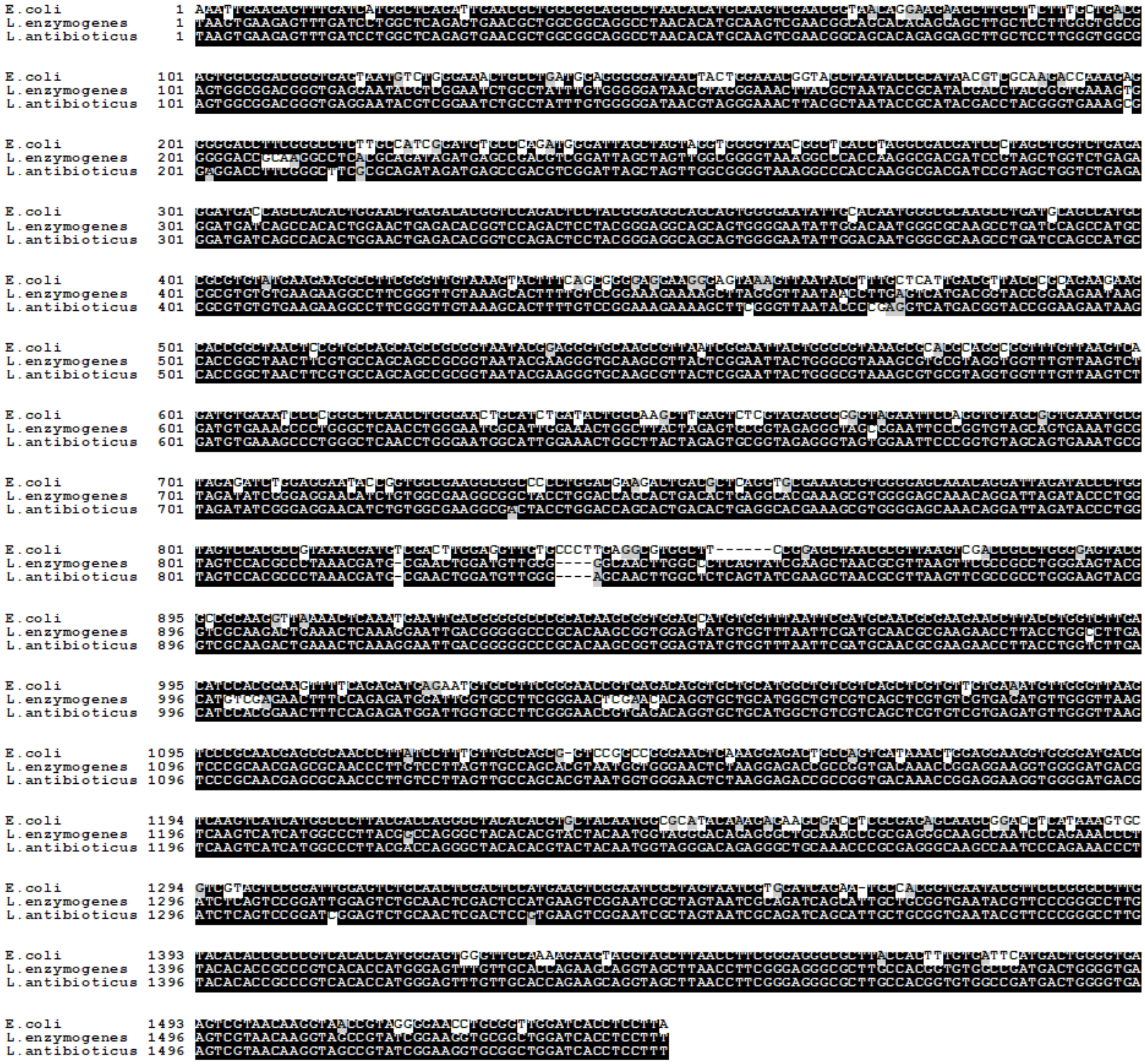

Fig. S1 Sequence alignment of 16S rRNA genes from *E. coli*, *L. enzymogenes*, and *L. antibioticus*. Conserved nucleotides are shaded black, similar ones are shaded grey. The DNA alignment was created by MUSCLE (https://www.ebi.ac.uk/Tools/msa/muscle/) and visualized using BOXSHADE (https://embnet.vital-it.ch/software/BOX_form.html).

Edgar, R.C. (2004). MUSCLE: multiple sequence alignment with high accuracy and high throughput. *Nucleic Acids Res.* 32:1792-1797.

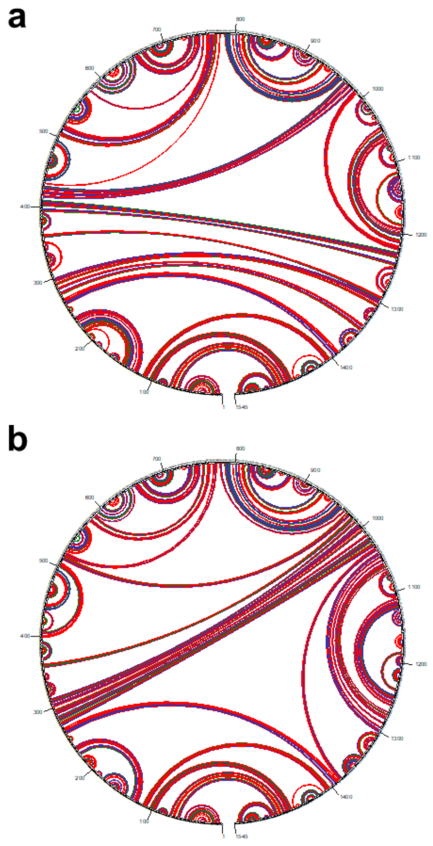

Fig. S2 Circle graphs representing putative secondary structures of 16S rRNAs from LeC3 (a) and LaATCC29479 (b). Circle graphs were generated with mfold program (http://unafold.rna.albany.edu/?q=mfold/RNA-Folding-Form) using default settings. Bases in 16S rRNA were equally placed around the circle in a clockwise fashion. G-C pairs are drawn in red arcs, A-U in blue, G-U in green, and other pairs (if present) in yellow. LeC3: *Lysobacter enzymogenes* strain C3; LaATCC29479: *Lysobacter antibioticus* strain ATCC29479.

Table S1 Primers used in this study

**Primer**	**Sequences (5' to 3')**
**Primers for identification of insertion sites**	
KAN-2 FP-1	ACCTACAACAAAGCTCTCATCAACC
KAN-2 RP-1	GCAATGTAACATCAGAGATTTTGAG
*Pvu*I-left	GAAAAACAGCATTCCAGGTATTAGA

*Pvu*I-right	AAGTTTATGCATTTCTTTCCAGACT
inv-1	ATGGCTCATAACACCCCTTGTATTA
inv-2	GAACTTTTGCTGAGTTGAAGGATCA
**Primers for confirmation of insertion sites**	
Tn5 KanF	TACACATCTCAACCATCATC
Tn5 KanR	ACACATCTCAACCCTGAAGC
16S rRNA IF-1	AGCTTTGCCGTTTCAGTCAT
16S rRNA IF-2	GCGGTTTCGATACGTGTT
16S rRNA IR	ACCTTCTACGGCGAACCTTT
23S rRNA IF-1	AAAGGTTCGCCGTAGAAGGT
23S rRNA IR-1	GTCGCGGATCGCGGGTTGCG
23S rRNA IF-2	AAGGTGACAGCCCTGTATGC
23S rRNA IR-2	CGGCGACTGCAGCCAGGCGA
rpoD IF	ATACCGTGTATTCGCCGATG
rpoD IR	TTGTGCTGCTCGTAATCGTC
purB IF	ATCTCGCTGGGCTATTTCAA
purB IR	GCAAGCGGGATCAGATTTC
mltB IF	CCTGCGAGCTGGATTTCTT
mltB IR	TCGCCAACTACTTCGTCAAG
**Primers for construction of vectors**	
mltB-F	CGTCaagcttCGGGGTGGAGTGAGAGGT
mltB-R	TCAtctagaCCTGCGAGCTGGATTTCTT
purB-F	CGTCaagcttGAGTTCGCCCATGCTGTC
purB-R	TCAtctagaACAATACGGTTACGGCGAAG
pMY205mPAG2-F	ACCGctcgagCCTTAAAGAAGCGTACTTTG
pMY205mPAG2-R	AAAAGGAAAAgcggccgcAAAAGTTTGACGCTCAAAGA
pMY205mPAG2-IF	GCGGGACTCTGGGGTTCG
Ec-F	AAAAGGAAAAgcggccgcAAATTGAAGAGTTTGATCAT
Ec-R	ACCGctcgagTAAGGAGGTGATCCAACCGC
LeLa-F	AAAAGGAAAAgcggccgcTAAGTGAAGAGTTTGATCCT
LeLa-R	ACCGctcgagAAAGGAGGTGATCCAGCCGC
**Primers for sequencing**	
M13For-40	GTTTTCCCAGTCACGAC
M13Rev-48	AGCGGATAACAATTTCACACAGGA

Table S2 List of Tn5 mutants with decreased resistance to spectinomycin in LeC3

No.	Insertion site	Accession	Gene	Description	Note
1	965,406	*GLE_6063*	*16S rRNA*	16S ribosomal RNA	
2	964,992	*GLE_6063*	*16S rRNA*	16S ribosomal RNA	
3	290,121	*GLE_6060*	*16S rRNA*	16S ribosomal RNA	
4	965,024	*GLE_6063*	*16S rRNA*	16S ribosomal RNA	
5	964,996	*GLE_6063*	*16S rRNA*	16S ribosomal RNA	
6	290,399	*GLE_6060*	*16S rRNA*	16S ribosomal RNA	
7	292,133	*GLE_6061*	*23S rRNA*	23S ribosomal RNA	
8	292,740	*GLE_6061*	*23S rRNA*	23S ribosomal RNA	
9	968,534	*GLE_6064*	*23S rRNA*	23S ribosomal RNA	
10	966,683	*GLE_6064*	*23S rRNA*	23S ribosomal RNA	
11	966,513	*GLE_6064*	*23S rRNA*	23S ribosomal RNA	
12	967,347	*GLE_6064*	*23S rRNA*	23S ribosomal RNA	
13	291,571	*GLE_6007*	*ala*	tRNA-Ala	
14	965,828	*GLE_6054*	*ala*	tRNA-Ala	
15	289,781	*GLE_0265 & GLE_6060*	*tyrS & 16S rRNA*	tyrosyl-tRNA synthetase & 16S ribosomal RNA	IGR
16	4295232	*GLE_3804*	*tuf*	translation elongation factor Tu	
17	408,359	*GLE_0368*	*rpoD*	RNA polymerase primary sigma factor	
18	5,029,914	GLE_4483	*mltB*	lytic murein transglycosylase B	
19	3,056,168	*GLE_2716*	*purB*	Adenylosuccinate lyase	

LeC3: *Lysobacter enzymogenes* strain C3, gene bank accession no. CP013140; IGR: Tn5 transposon was inserted into the intergenic region (IGR) in LeC3 genome.

Table S3 Known mutations in the spectinomycin-resistant 16S rRNA genes

**Resistance mutation (helix number)**	**Organism**	**Reference**
C1063U (helix 34)	Metagenome	(Miyazaki and Kitahara 2018)
G1064C (helix 34)	*Neisseria* spp.	(Wrather and Koenning 2006)
C1066U	*E. coli*, *S. enterica*	(Johanson and Hughes 1995; O'connor and Dahlberg 2002)
A1191G (helix 34)	*Borrelia burgdorferi*, *Chlamydia* spp.	(Binet and Maurelli 2005; Criswell et al. 2006)
C1192U (helix 34)	*E. coli*, *B. burgdorferi*, *Salmonella enterica*, *Chlamydia* spp.	(Binet and Maurelli 2005; Criswell et al. 2006; O'connor and Dahlberg 2002; Sigmund et al. 1984)
C1192G (helix 34)	*Chlamydia* spp.	(Binet and Maurelli 2005)
G1193C (helix 34)	*Chlamydia* spp.	(Binet and Maurelli 2005)
U1183C (helix 38)	Metagenome	(Miyazaki and Kitahara 2018)
U1189C	Metagenome	(Miyazaki and Kitahara 2018)

Resistance mutation number was given using nucleotide position in the 16S rRNA sequence of *Escherichia coli*.
